# Adhesive strength in cementing fiberglass posts using different adhesive systems

**DOI:** 10.4317/jced.62131

**Published:** 2025-04-01

**Authors:** Guilherme Do Amaral, Karina Andrea Novaes Olivieri, Rafael Pino Vitti, Clovis Stephano Pereira Bueno, William Cunha Brandt, Milton Edson Miranda

**Affiliations:** 1 Faculdade São Leopoldo Mandic; 2 Universidade Santo Amaro

## Abstract

**Background:**

The objective of this work was to evaluate the influence of the Scotchbond MP and Clearfil SE adhesive systems on the bond strength in the push out test between the fiberglass post and the root canal.

**Material and Methods:**

This *in vitro* study involved 20 human canine teeth extracted over a 12-month period. For inclusion in the sample, upper canine teeth with fully formed apices and curvatures less than 45° were considered. Teeth containing a calcified canal, with radiographically visible double curvature or that had undergone previous endodontic treatment were excluded from the sample. After selection, surface prophylaxis was performed using an ultrasound insert, followed by smoothing with a Gracey curette. Endodontic treatments were performed by a calibrated operator, following a pre-established protocol. Next, preparation of the root canal began to receive the Whitepost DC number 2 fiberglass post by removing 2/3 of this space. Next, the posts were prepared to be cemented, they were cleaned with 70% alcohol and conditioned with 37% phosphoric acid before receiving two layers of silane. From that moment on, the elements were randomly divided into two groups, according to the adhesive system, with RelyX ARC cement being used: Group SCB: Scotchbond MP and Group CFL: Clearfil SE. After cementing the fiber posts, the test specimens were prepared for mechanical testing using the push out test on the Universal Testing Machine (EMIC). Multiple comparisons were performed using the Tukey test. Statistical calculations were conducted adopting a significance level of 5%.

**Results:**

The two-factor analysis of variance demonstrated that there was no statistically significant interaction between the factors studied, however the use of the SCB Group adhesive system produced higher RU values.

**Conclusions:**

It was concluded that greater bond strength was obtained between resin cement and root dentin when ScotchBond adhesive was used and there were no differences in RU in the different root portions.

** Key words:**Fiberglass posts, Adhesive systems, Tensile strength, Cementation, Dental prosthesis.

## Introduction

Cementation of intraradicular posts plays a crucial role in the rehabilitation of endodontically treated teeth, enabling the reconstruction of the lost coronal portion. In the current context of restorative dentistry, fiberglass posts emerge as a promising alternative to traditional metal posts, offering significant advantages in terms of aesthetics, in addition to biocompatibility and the ability to adhere to the cement and the cement to the dental tissue. In this context, the type of dentin adhesives in the cementation of fiberglass posts has been the subject of increasing interest and research, aiming to improve the retention and durability of intraradicular restorations (1-12).

Dentin adhesives play a fundamental role in the cementation of fiberglass posts, facilitating adhesion between the restorative material and the remaining tooth structure. Through a combination of adhesive agents, solvents, and resin monomers, these materials promote the formation of a sTable hybrid interface between dentin and resin, improving retention and reducing the risk of marginal infiltration and fractures. In addition, dentin adhesives offer sealing and bonding properties that help prevent the penetration of microorganisms and fluids into the tooth-restoration interface, contributing to the longevity and integrity of the restorative treatment (7,10,13).

Understanding the principles of adhesion and application of dentin adhesives in the cementation of glass fiber posts is essential to optimize clinical results and minimize complications. Proper selection of the adhesive system, preparation of the dentin surface, and application technique are critical factors that influence the efficacy and durability of intraradicular post cementation. Furthermore, considerations such as the anatomy of the root canal, the presence of caries or endodontic lesions, and the quality of the remaining tooth must be carefully evaluated to ensure reliable adhesion and a functional and esthetic restoration (14-25).

The study on the adhesion of fiberglass posts is relevant to clinical practice and the advancement of restorative dentistry for several reasons. The research aims to evaluate the influence of different adhesive systems on the bond strength in the push-out test between the fiberglass post and the root canal. The null hypothesis established is that there will be no influence on the bond strength in relation to the adhesive system used, regardless of the root region, and that the different root regions would have the same RU values.

## Material and Methods

-Materials

Sample

The study was approved by the Local Research Ethics Committee (Appendix A), with opinion number 5,879,836 and CAAE 60615522 2 0000 5374. All patients who donated their teeth for research signed an Informed Consent Form (Appendix B).

The sample for this laboratory study consisted of 20 human canine teeth extracted over a 12-month period 23.

The teeth were stored in a 1% thymol solution immediately after extraction. For inclusion in the sample, upper canine teeth with fully formed apices and curvatures less than 45° were considered (26). Teeth containing a calcified canal, with a radiographically visible double curvature or submitted to previous endodontic treatment were excluded from the sample.

-Method

Sample preparation

The selected samples underwent surface prophylaxis. For this purpose, a needle holder (ICE, São Paulo, Brazil) was used to remove coarser materials on the root surface, such as the bone plate. Then, an H3 ultrasonic insert (Obtura Spartan, Algonquin, USA) was used to remove tartar and, finally, a Gracey curette number 5-6 (J&J Instruments, Linden, USA) was used to smooth the root surface. Endodontic treatments were performed by a single, previously calibrated operator, following a pre-established protocol.

Endodontic technique

Initially, an X-ray was taken and the teeth were accessed using an Eletromatic Kavo electric motor with a 1:5 multiplier (Kavo do Brasil Ind. Com. Ltda., Joinville, Brazil) and spherical diamond burs numbered 1012 (Komet, Santo André, Brazil). Once the pulp chamber was accessed, the 1012 bur was replaced by the 3082 bur to create the contour shape and complete the coronal access.

The canals were explored with manual K-files numbered 10 and 15 (Dentsply Maillefer, Ballaigues, Switzerland), using oscillatory movements and taking the instrument 2 mm below the initial length of the tooth. Next, odontometry was performed visually and with the aid of a CDR Elite digital sensor (FONA Schick, São Paulo, Brazil) to establish the instrumentation length of each sample. The working length was set at 1 mm below the actual length of the tooth.

The ProTaper Ultimate rotary system (Dentsply Maillefer, Ballaigues, Switzerland) was used to prepare the root canal, which contains files 16.02, 20.04, 20.07, 25.08, and 30.09. The endodontic files were used by applying three in-and-out movements with a gentle brushing action when removing the instrument from the canal. After each instrument was used, the canal was irrigated with 5 mL of a 2.5% sodium hypochlorite solution (Fórmula e Ação, São Paulo, Brazil).

After the chemical-mechanical preparation, the smear layer was removed with 17% EDTA (Fórmula e Ação, São Paulo, Brazil) to proceed with the obturation. The filling was performed using the single cone technique using calibrated FM gutta-percha cones (Tanariman Industrial Ltda., Amazonas, Brazil) and Endomethasone N endodontic cement (Septodont, Cedex, France), which is classified as a zinc oxide and eugenol-based cement.

Intraradicular preparation

Next, the root canals were prepared to receive the Whitepost DC fiberglass post number 2 (Dentscare Ltda, Santa Catarina, Brazil) by removing 2/3 of this space. To remove the filling, a Gates Glidden drill (Komet Brasil, São Paulo, Brazil) number 3 was used, followed by a drill number 2 from the manufacturer’s own post system (Dentscare Ltda, Santa Catarina, Brazil).

The posts were then prepared to be cemented: they were cleaned with 70% alcohol (Prolink Indústria Química Ltda, São Paulo, Brazil), conditioned with 37% Condac phosphoric acid (Dentscare Ltda, Santa Catarina, Brazil) for 15 seconds, and washed in running water for 1 minute. The posts were then dried with a gentle air jet and silanized with Prosil (Dentscare Ltda, Santa Catarina, Brazil). Two layers of silane were applied with a one-minute interval between them and a light jet of air was used for 20 seconds after each layer.

Experimental groups

From this point on, the samples were randomly divided into two groups, according to the adhesive system: Group SCB – Scotchbond MP (3M ESPE, Minnesota, USA) and Group CFL – Clearfil SE (Kuraray Noritake Dental Inc., Okayama, Japan).

In Group SCB, after silanization of the posts, a layer of Scotchbond adhesive was used followed by photopolymerization with a GranValo device (Ultradent Products, Inc., Indaiatuba, Brazil). In Group CFL, a layer of Clearfil adhesive was applied. In both groups, the photoactivation of the adhesive on the post was in Standard mode (1000 mW/cm2) for 20 seconds per side, totaling 80 seconds.

After preparation of the posts, the preparation of the canals was returned to perform cementation of the posts. In the SCB Group, conditioning was performed with 37% Condac phosphoric acid (Dentscare Ltda, Santa Catarina, Brazil) in the root canal for 15 seconds. The canal was then washed for one minute and dried with an endodontic suction kit (Ultradent Products, Inc., Indaiatuba, Brazil) and absorbent paper cones (Tanariman Industrial Ltda, Amazonas, Brazil). Next, two layers of ScotchBond primer were applied, rubbing the microapplicator against the walls (active form), waiting for a period of one minute between them. A gentle air jet was used before applying the Scotchbond system adhesive layer, which was applied with the microapplicator without rubbing the walls. In the CFL Group, phosphoric acid was not applied since the primer is acidulated. Two layers of Clearfil system primer were applied actively with a one-minute interval between them and after applying a gentle air jet, the Clearfil adhesive was applied. Both the primer and the adhesive of the systems used were applied in the same way

Both groups had the canal adhesive photoactivated in Standard mode for 60 seconds after removing excess with the endodontic suction kit.

Post cementation

Then, the Relyx ARC cement (3M do Brasil Ltda, São Paulo, Brazil) was manipulated on a glass plate and inserted into the root canal together with the fiberglass post, observing the length. According to the manufacturer’s standards, the set was photoactivated in Standard mode for 40 seconds on each side, totaling 200 seconds per sample.

Sectioning of samples

The twenty samples were sectioned perpendicularly to the cementoenamel junction with a carborundum disk under refrigeration to create specimens. The roots were then fixed with sticky wax on acrylic plates and subsequently sectioned perpendicularly to their long axis using the Extec Dia Wafer Blade 4” x .012 x ½ (102 mm X 0.3 mm X 127 mm) disk that was coupled to the metallographic trimmer - Isomet 1000, in order to obtain samples with 2 mm thickness of the cervical, middle and apical thirds.

The samples were immersed in distilled water and stored in the ECB 1.3 digital bacteriological oven (Odontobrás, Ribeirão Preto, Brazil) at 37°C for 72 hours.

Mechanical test

The specimens were subjected to the push-out test in the Universal Testing Machine (EMIC), being mounted so that the loads were applied in the apicocervical direction at a speed of 0.5 mm/min until the pin was dislodged. The peak force at the point where the pin moved out of the specimen segment will be considered as the bond strength of the pin/root of the cementation and this force will be recorded in KgF. The fractured specimens were measured with the aid of a digital caliper (Absolute/Digimatic Mitutoyo) to convert the results into megapascals (MPa).

The force was recorded in kilograms/force at the moment of fracture of the specimens by the program coupled to the testing machine (TESC version 3.01). The software also recorded the stress values in MPa corresponding to the conversion of the values into KgF.

Statistical analysis

Prior to the analyses, the bond strength data were assessed for normality using the Shapiro-Wilk test. They were then subjected to two-way analysis of variance. The study factors were the type of adhesive used (Clearfil or ScotchBond) and the root region (Cervical, Middle or Apical). Multiple comparisons were performed using the Tukey test. Statistical calculations were performed using a significance level of 5% (α = 0.05) using the SigmaPlot 12.0 program (Systat Software Inc., San Jose, California, USA).

## Results

The two-way analysis of variance demonstrated that there was no statistically significant interaction between the factors studied (*p*=0.906). The “region” factor was also not statistically significant (*p*=0.054). However, the “adhesive” factor demonstrated differences between the groups analyzed (*p*=0.003).

 Table 1 shows the mean values and standard deviation of bond strength between resin cement and root dentin in different regions when different adhesives were used.

Analysis of the results demonstrated that the use of ScotchBond adhesive led to higher bond strength values between resin cement and root dentin.

**Figure 1:**
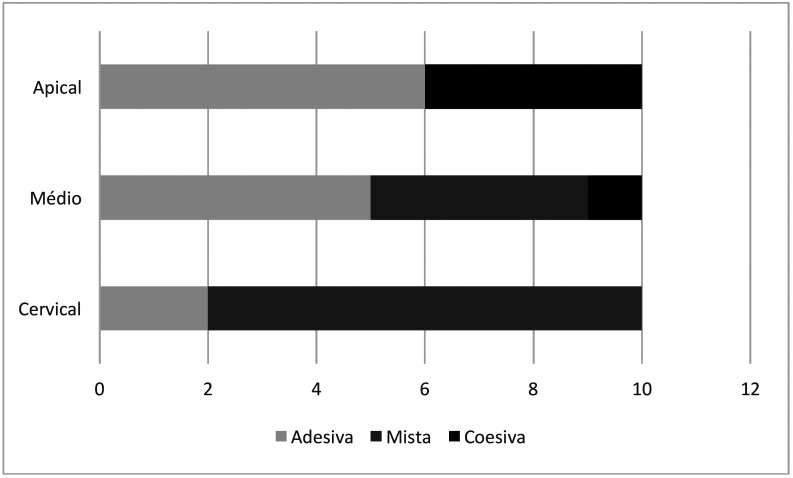
Failure pattern of samples with Clearfil adhesive. Source: Own authorship.

**Figure 2:**
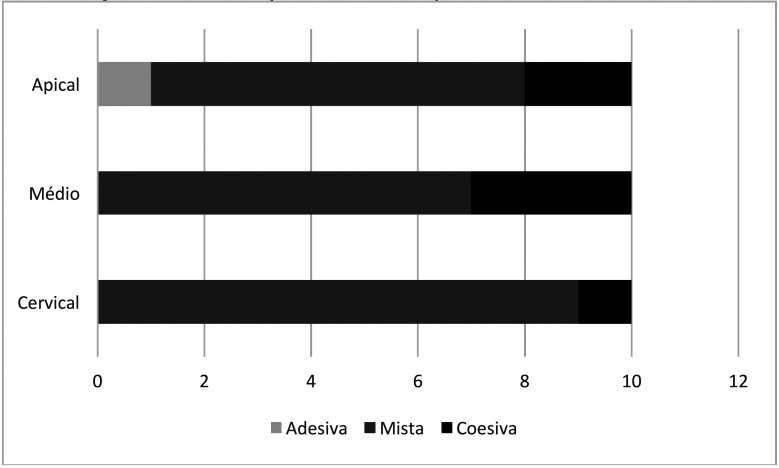
Failure pattern of samples with ScotchBond adhesive. Source: Own authorship

Figures 1 and 2 show the failure pattern when different adhesives were used to bond the fiber post, cement and root dentin.

## Discussion

Stated that the loss of retention of fiber posts is one of the main causes of treatment failures (7). This can be explained by the difficulty in obtaining stable adhesion in intraradicular dentin compared to coronal dentin. The endodontic technique, intraradicular preparation, adhesive materials, handling and activation method are some of the variables that impact the cementation process. Cited other complications (3,5). These studies developed the detrimental relationship between sodium hypochlorite and EDTA solutions with the adhesive capacity of the materials. In 2018, found that irrigating solutions affected adhesives applied in self-etching mode more than conventional adhesives (25). In the present study, it is highlighted that all samples used the same endodontic technique protocol, although we used such solutions.

Observed that the longer the time interval between root canal obturation and post cementation (9,14), the worse the adhesion becomes, regardless of the portion of the canal. The authors also observed that cements containing eugenol impaired adhesion by themselves, as did (1,18). In the present study, we performed intraradicular preparation and installation of the fiberglass post immediately after obturation; however, we used a cement that contains eugenol in its formulation. Zinc oxide and eugenol-based cements are popular in the market due to their antimicrobial activity, low cost, ease of handling, high radiopacity, and long setting time (26-30). In their work (11) observed that intracanal medication, used between endodontic treatment sessions, such as calcium hydroxide, interferes with adhesion.

Found no differences between the thirds of the root canal when examining adhesive strength (6). The current study also found no differences. However, these results diverge from those of (10), who observed that light transmission through glass fiber posts decreased as depth increased and that polymerization of the resin cement decreased significantly beyond a depth of 5 mm. Irradiance along the post decreased exponentially, which led to insufficient polymerization of the dual-cure resin cement around these posts in the most apical region. As well as the study (24), who found lower push-out values in the apical region, regardless of the protocol used. While (21) observed that a 5-minute delay for photoactivation significantly increased the bond strength of all cements evaluated, highlighted that the type of resin cement will have a greater impact on the result (28), since self-adhesive cement presents higher bond strength values in the cementation of fiberglass posts in deeper areas.

Compared conventional and self-etching adhesives for cementing fiberglass posts associated with a dual resin cement (12). They observed that conventional systems produced the best formation of the hybrid layer, with results comparable to those of (7). These results are in line with the current study, which observed greater bond strength between resin cement and root dentin when using ScotchBond adhesive in conjunction with RelyX ARC, but differ from the results of who demonstrated that a self-etching adhesive resulted in a higher average bond strength value compared to the conventional adhesive system (2) and who did not observe superiority of one of the materials (24).

RelyX ARC (conventional) and RelyX U200 (self-adhesive) cement in their research and observed that RelyX ARC obtained higher polymerization shrinkage and degree of conversion values and that these values were higher in the cervical third compared to the apical third (23). Observed that the association of RelyX ARC cement presented greater bond strength when associated with the conventional adhesive technique (29). This is a possible justification for the results of the present study, which also obtained higher values when the conventional adhesive was used.

Not using phosphoric acid inside the root canal has advantages, but the durability of the bond needs to be proven by long-term clinical studies (16). Therefore, the use of fiber posts is an alternative to metal posts in the restoration of endodontically treated teeth. It is worth highlighting the need for long-term clinical trials to reinforce this statement.

Carried out a study with the objective of evaluating the immediate and long-term bond strength through the push-out test of fiberglass posts cemented with two types of double-activation resin cements, conventional and self-adhesive in the three root thirds (cervical, middle, apical) (13). The posts (Reforpost No. 3, Angelus) were cleaned with 37% phosphoric acid (30 s) and silane (1 min). Thirty single-rooted roots were divided into two groups (n = 15) according to the type of resin cement: ARC dual cement (RelyX ARC - 3M ESPE) associated with the conventional three-step adhesive system (Adper ScotchBond Multipurpose Plus - 3M ESPE) or self-adhesive cement (RelyX U200 - 3M ESPE). Cementation was performed according to the manufacturer’s instructions. After 48 hours, the roots were sectioned transversely into different thirds, obtaining cervical, middle and apical root slices, and randomly divided into two groups, according to the water storage period (48 hours or 180 days) for the push-out test. The analysis of the results did not demonstrate significant interactions between the three factors analyzed: type of cement X time X root thirds (*p*=0.716). Regarding the fracture mode, there was a higher prevalence of adhesive fractures between cement and dentin, except for the RelyX ARC group, tested in the first 48 hours, in the cervical third, which presented predominantly adhesive fractures, between the post and the cement (55%). In the RelyX U200 cement, in the samples stored for 180 days, in the middle and apical third, cohesive fractures prevailed 40 and 45%, respectively. Therefore, the type of cement, water storage and depth did not influence the bond strength of glass fiber posts to root dentin. Evaluated the degree of conversion (GC) and adhesion of resin cements to glass fiber posts in different regions of the root canal. Single-rooted teeth were sectioned at the cementoenamel junction (CEJ), endodontically treated and prepared to a depth of 8 mm for fixation of glass fiber posts (RelyX Fiber Post, 3M ESPE) and randomly divided into two groups according to the resin cement: cements with methacrylate and phosphate acid monomers Group ML (Multilink Automix, Ivoclar Vivadent) and Group RXU (RelyX Unicem 2 Automix, 3M ESPE). The roots were sectioned into 2-mm-thick slices (n=3 per root), respectively, at 1, 3 and 5 mm apically to the CEJ. The GC was evaluated by Micro-Raman Spectroscopy, the bond strength by push-out and the fracture mode by optical microscopy. Regardless of the type of cement, the mean bond strength results (MPa) were significantly higher in the coronal slices than in the apical ones (*p*=0.002). The type of cement and the depth of the root canal affected the degree of conversion, however the interaction of the factors was not significant. The GC was higher for Multilink Automix than for RelyX Unicem 2 Automix. The RXU group presented a greater number of adhesive fractures between cement and dentin, while in the MX group the highest incidence of adhesive fractures was between the pin-cement. Considering the push out, GC and fracture mode tests, cements with methacrylate and phosphate acid monomers should be preferred for cementing glass fiber posts when compared to cements that require acid etching. In the present study, we did not observe a statistically significant difference between the results of bond strength of the coronal and apical slices. However, the adhesives studied showed differences between the groups analyzed.

Clinical studies can provide valuable information, but it is important to consider the importance and impact of laboratory studies in scientific research. Work carried out in laboratories is essential for the creation of hypotheses and the creation of new methods.

## Conclusions

It was concluded that greater bond strength between resin cement and root dentin was obtained when ScotchBond adhesive was used and there were no differences in RU in the different root portions.

## Figures and Tables

**Table 1:** Mean and standard deviation (MPa) of the bond strength values.

Adhesivo	Apical	Medium	Cervical	Medium
Clearfil	16,0 (13,1)	23,6 (12,4)	29,9 (23,9)	23,6 (17,6) b
ScotchBond	30,2 (12,7)	33,5 (10,3)	42,3 (15,8)	35,3 (13,7) a

Source: Own authorship

## Data Availability

The datasets used and/or analyzed during the current study are available from the corresponding author.

## References

[1] Altmann AS, Leitune VC, Collares FM (2015). Influence of eugenol-based sealers on push-out bond strength of fiber post luted with resin cement: systematic review and metaanalysis. J Endod.

[2] Barcellos DC, Huhtala MFRL, Silva MA, Gomes APM, Franco LT (2014). Influence of adhesive system in bond strength of fiber glass posts to radicular dentin using dual cure resin cement. Brazilian Dental Science.

[3] de Deus G, Filho EDG, Ferreira CM, Filho TC (2002). Intratubular penetration of root canal sealers. Pesqui Odontol Bras.

[4] Diaz-Arnold AM, Vargas MA, Haselton DR (1999). Current status of luthing agents for fixed orthodontics. J Prosthet Dent.

[5] Donnermeyer D, Vahdat-Pajouh N, Schäfer E, Dammaschke T (2019). Influence of the final irrigation solution on the push-out bond strength of calcium silicate-based, epoxy resin-based and silicone-based endodontic sealers. Odontology.

[6] Giachetti L, Scaminaci Russo D, Baldini M, Bertini F, Steier L, Ferrari M (2012). Push out strength of translucent fiber posts cemented using a dual-curing technique or light-curing self-adhering material. Int Endod J.

[7] Goracci C, Ferrari M (2011). Current perspectives on post systems: a literature review. Aust Dent J.

[8] Grandini S, Goracci C, Monticelli F, Borrachini A, Ferrari M (2005). SEM evaluation of the cement layer thickness after luting two different posts. J Adhes Dent.

[9] Hagge MS, Wong RDM, Lindemuth JS (2002). Retention strengths of five luting cements on prefabricated dowels after root canal obturation with a zinc oxide/eugenol sealer: 1. Dowel space preparation/cementation at one week after obturation. J Prosthodont.

[10] Ho YC, Lai YL, Chou IC, Yang SF, Lee SY (2011). Effects of light attenuation by fiber posts on polymerization of a dual-cured resin cement and microleakage of postrestored teeth. J Dent.

[11] Lee BS, Lin YC, Chen SF, Chen SY, Chang CC (2014). Influence of calcium hydroxide dressing and acid etching on the push-out bond strengths of three luting resins to root canal dentin. Clin Oral Investig.

[12] de Melo RM, Bottino MA, Galvão RKH, Soboyejo WO (2012). Bond strengths, degree of conversion of the cement and molecular structure of the adhesive-dentine joint in fibre post restorations. J Dent.

[13] Mendes M, França FMG, Basting RT, Turssi CP, Amaral FL (2016). Long term bond strength of fiber posts cement to dentin with self-adhesive or conventional resin cements. J Adhes Sci Technol.

[14] Menezes MS, Queiroz EC, Campos RE, Martins LRM, Soares CJ (2008). Influence of endodontic sealer cement on fibreglass post bond strength to root dentine. Int Endod J.

[15] Mitchell CA (2000). Selection of materials for post cementation. Dent Update.

[16] Mondelli J (1998). Técnicas restauradoras para dentes com tratamento endodôntico. Rev Dent Rest.

[17] Morgano SM, Rodrigues AH, Sabrosa CE (2004). Restoration of endodontically treated teeth. Dent Clin North Am.

[18] Mosharraf R, Zare S (2014). Effect of the type of endodontic sealer on the bond strength between fiber post and root wall dentin. J Dent (Tehran).

[19] Namoratto LC, Ferreira RS, Lacerda RAV, Sampaio Filho HR, Ritto FP (2013). Cimentação em cerâmica: evolução dos procedimentos convencionais e adesivos. Rev. Bras. Odontol.

[20] Pereira JR, Martins LCN, Paula VG, Ghizoni JS, May NB, Pamato S (2012). Análise de resistência à tração de pinos de fibra de vidro cimentados com diferentes cimentos de ionômero de vidro através do teste pull-out. RFO UPF.

[21] Pereira RD, Valdívia AD, Bicalho AA, Franco SD, Tantbirojn D, Versluis A (2015). Effect of photoactivation timing on the mechanical properties of resin cements and bond strength of fiberglass post to root dentin. Oper Dent.

[22] Phrukkanon S, Burrow MF, Tyas MJ (1999). The effect of dentin location and tubule orientation on the bond strengths between resin and dentin. J Dent.

[23] Pulido CA, De Oliveira Franco AP, Gomes GM, Bittencourt BF, Kalinowski HJ, Gomes JC (2017). An in situ evaluation of the polymerization shrinkage, degree of conversion, and bond strength of resin cements used for luting fiber posts. J Prosthet Dent.

[24] Rodrigues RV, Sampaio CS, Pacheco RR, Pascon FM, Puppin-Rontani RM, Giannini M (2017). Influence of adhesive cementation systems on the bond strength of relined fiber posts to root dentin. J Prosthet Dent.

[25] Shafiei F, Mohammadparast P, Jowkar Z (2018). Adhesion performance of a universal adhesive in the root canal: Effect of etch-and-rinse vs. self-etch mode. PLoS One.

[26] Schneider SW (1971). A comparison of canal preparations in straight and curved root canals. Oral Surg Oral Med Oral Pathol.

[27] Silva RAT, Coutinho M, Cardozo PI, Silva LA, Zorzatto JR (2011). Conventional dual-cure versus self-adhesive resin cements in dentin bond integrity. Journal of applied oral Science.

[28] Soares CJ, Pereira JC, Valdivia ADCM, Novais VR, Meneses MS (2012). Influence of resin cement and post configuration on bond strength to root dentine. Int Endod J.

[29] Souza-Junior EJ, Bueno VCPS, Dias CTS, Paulillo LAMS (2010). Effect of endodontic sealer and resin luting strategies on pull-out bond strength of glass fiber posts to dentin. Acta Odontol Latinoam.

[30] Tedesco M, Chain MC, Bortoluzzi EA, Garcia LFR, Alves AMH, Teixeira CS (2018). Comparison of two observational methods, scanning electron and confocal laser scanning microscopies, in the adhesive interface analysis of endodontic sealers to root dentine. Clin Oral Investig.

